# Hemoadsorption and continuous venovenous hemodiafiltration in the management of paraquat poisoning during pregnancy: A case report

**DOI:** 10.1016/j.toxrep.2023.11.003

**Published:** 2023-11-07

**Authors:** David A. Ballesteros, Daniel R. Santiago, Maria E. Barrera, Andrea C. Mantilla

**Affiliations:** a Nephrology Department, San José University Hospital, Popayán, Cauca, Colombia; b Department of Internal Medicine, University of Cauca, Popayán, Cauca, Colombia

**Keywords:** Paraquat, Poisoning, HA-230, Case report, hemoadsorption, hemodiafiltration

## Abstract

We describe the case of a mother in the second trimester of pregnancy with severe paraquat poisoning who ended her pregnancy at term and a healthy newborn. Management was initiated after 34 h of paraquat administration with the HA-230 hemoadsorption cartridge, followed by continuous venovenous hemodiafiltration for 120 h, in addition to cyclophosphamide and methylprednisolone. There was no evidence of adverse effects associated with treatment or extracorporeal therapy, and maternal and fetal well-being was maintained during the 26 days of hospitalization and at the end of pregnancy. This case treated with hemoadsorption and hemodiafiltration for paraquat poisoning during pregnancy is one of the few procedures reported in the literature and can be used as a guide for the management of subsequent cases.

## Highlights

• Paraquat poisoning is associated with high mortality, cases in pregnant women are rare, the treatment is challenging, raises ethical issues, and evidence is scant.
• Hemoadsorption lowers paraquat levels in the blood and has been associated with improved survival.
• Hemodiafiltration removes cytokines generated by the inflammatory response associated with paraquat poisoning.
• The combined use of Hemoadsroption, hemodiafiltration, cyclophosphamide and steroids can maintain maternal and fetal well-being.

## Graphical Abstract

**Figure:**
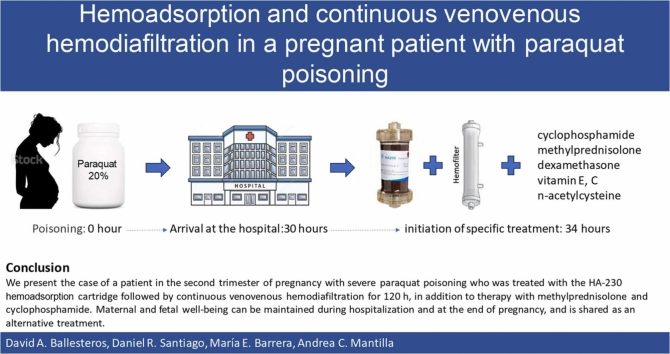

## 1 Introduction

Paraquat (1,1′-dimethyl-4,4′-bipyridyl dichloride) is a herbicide belonging to the bipyridyl family. It is found in liquid form at a concentration of 20% for agricultural use and is classified as a highly toxic substance after ingestion. It is associated with high mortality rates [1], making it an important public health concern in developing countries [2]. The high lethality of paraquat is due to its inherent toxicity and lack of a specific antidote. There are no widely accepted guidelines for the treatment of patients with paraquat poisoning, and treatment focuses on immunomodulation (corticosteroids and cyclophosphamide), antioxidants (N-acetylcysteine and vitamin C), extracorporeal support (Hemoadsoption and hemodiafiltration), and antifibrotic drugs (pirfenidone) [1]. Previous studies have suggested that hemoadsorption is associated with better survival in patients poisoned with paraquat, which is a novel option because the high rate of elimination of paraquat from the blood is associated with longer survival [3].

Paraquat poisoning in pregnant women and their fetuses is difficult to treat and poses challenges due to various ethical issues; therefore, it is rarely reported in literature. We present a case of paraquat poisoning in a pregnant woman treated with Hemoadsroption and hemodiafiltration.

## 2 Case report

**Fig. 1:**
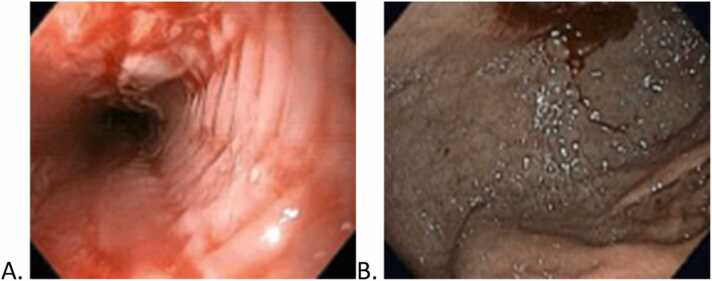
Esophagogastroduodenoscopy that showed caustic esophagitis, Zargar 2A (A) and erosive antrocorporal gastritis (B.).

A 23-year-old pregnant female was admitted at 23.2 weeks from a rural area in Colombia with a history of three previous suicide attempts two years prior to admission due to sodium fluoroacetate. The patient consulted at the first level after taking approximately 25 ml of 25% paraquat with suicidal intentions, gastric lavage was performed with saline solution and activated charcoal and she was referred to our institution. She was admitted to the hospital 30 h later because of transportation difficulties, with the following vital signs: blood pressure, 116/72 mmHg; heart rate, 75 beats per minute; respiratory rate, 18 breaths per minute; temperature, 36 °C; oxygen saturation, 95% FiO2, 21%; and physical examination revealed several ulcers on the oral mucosa. She was transferred to the intensive care unit (ICU) with an APACHE II score of 7 points. On the second day of hospitalization, obstetric ultrasound was performed with a report of a single fetus, with an estimated weight of 589 g at the 61st percentile of growth, with symmetrical, harmonious growth profile and normal body segment ratios for gestational age, placenta at the posterior fundal level, grade I/III maturation, with a thickness of 20,2 mm and Doppler of uterine arteries with normal percentile for gestational age. The mother had oral and pharyngeal burns, so upper digestive tract endoscopy showed caustic esophagitis Zargar 2 A and erosive antrocorporal gastritis (Fig. 1).

The patient was treated according to the institutional protocol [1], which involved administering methylprednisolone 1 g every 24 h during the first three days. A medical meeting was held to assess the risk/benefit and it was determined to start cyclophosphamide 900 mg/day during the second and third days of hospitalization. Additionally, the lung maturation protocol was initiated at 24 weeks with betamethasone 12 mg every 24 h for two doses. 34 h after paraquat intoxication, Hemoadsroption was performed with a Jafron HA-230 cartridge for six hours and continuous venovenous hemodiafiltration (CVVHD) was continued for 120 h, requiring three oXiris filters and one ST-150 filter, due to the patient's clinical condition, no systemic anticoagulation or regional anticoagulation of the hemodiafiltration circuit was used. After suspending therapy, presented acute kidney injury KDIGO 2 managed with intermittent hemodialysis on two occasions, patient recovered renal function and high-flow catheter was removed, laboratory description was performed during the first five days of treatment and at discharge in Table 1. The patient presented with hypokalemia, hypomagnesemia, and hypophosphatemia that required replacement during his stay in the intensive care unit, received parenteral nutrition for 15 days, and management continued according to the institutional protocol with ascorbic acid, n-acetylcysteine, and vitamin E for 21 days in gynecology rooms. On the last day of hospitalization, they performed an obstetric ultrasound for control by maternal-fetal medicine with a report of pregnancy of 27.4 weeks, estimated fetal weight 1005 g ± 18.1th percentile (Hadlock) for gestational age, posterior fused placenta grade II/III of maturation, amniotic fluid index:14.94 cm (Phelan technique) and discharged after 26 days of hospitalization, and was followed up by telephone. She reported that her pregnancy ended without complications in another institution and three weeks postpartum, the newborn was healthy, she has a psychiatric follow-up.

**Table 1:** Laboratories during the first five days of treatment and at discharge.

Laboratories	Day 0	Day 1	Day 2	Day 3	Day 4	Day 5	Discharge
Creatinine (mg/dl)	1,24	1,31	1,39	1,21	1	0,92	0,53
BUN (mg/dl)	10	14	16	16	20	19	13
Sodium (mEq/L)	134	135	136	137	139	137	137
Potassium (mEq/L)	3,5	3,1	3,5	4,6	4,2	3,7	3,8
Chlorine (mEq/L)	102	103	103	103	107	105	103
Hemoglobin (g/dl)	10,7	10,4	9,9	8,5	8,3	8	9,4
leukocytes (x10^3/ul)	12,5	13,1	10,7	5,8	5,7	4,9	10,3
Plaquetas (x10^3/ul)	299	145	110	83	67	55	355
AST (U/l)	37	45	40	48	-	-	-
ALT (u/L)	16	17	21	44	-	-	-
Total bilirubin (mg/dl)	1	0,7	0,7	0,6	-	-	-
pH	7,45	7,44	-	7,52	-	-	-
PaCO2 (mmHg)	30	28	-	25	-	-	-
PaO2 (mmHg)	72,1	77	-	88	-	-	-
PaO2/FiO2 (mmHg/%)	3,55	3,92	-	4,46	-	-	-

Abbreviations: BUN: blood urea nitrogen; PaO2: partial pressure of oxygen; PaCO2: partial pressure of carbon dioxide; AST: aspartate aminotransferase; ALT: Alanine aminotransferase. -: Denotes values were not obtained.

Notes: Day 0: corresponds to the laboratories before the start of treatment.

## 3 Discussion

Paraquat is a highly toxic herbicide for humans. It rapidly enters the body through the digestive tract and is distributed in the liver, kidneys, lungs, and other organs. The volume of distribution of paraquat is 1.2–1.6 L/Kg, it is excreted mainly through the urine, with a rate of 82.9% in 48 h [2]. The mean distribution half-life of paraquat in plasma is 5 h, whereas the mean elimination half-life is 84 h, which can be explained by three-compartment theory [3], [4]. This theory is not alien to obstetric patients, similar concentrations of paraquat have been found in the mother, fetus and amniotic fluid, which suggests that the ability to eliminate the toxin by the fetal organs develops at the end of pregnancy [5], [6].

Paraquat poisoning by oral ingestion in pregnant women is rare; it has previously been shown that it can cross the placental barrier and cause fetal death [7]. We present the case of a patient in the second trimester of pregnancy with severe paraquat poisoning; hemoadsorption was performed followed by CVVHD, a total of 120 h of treatment with the intention of removing the greatest amount of paraquat and cytokines, based on pharmacokinetic aspects, together with the use of steroids and cyclophosphamide despite having pregnancy category D, the potential benefits outweighed the risks. The clinical efficacy of Hemoadsorption in paraquat poisoning is based on the early elimination of the toxicant from the blood, and clinical studies have shown that the best time for PH is 2–4 h after ingestion [8]. In the present case, therapy was started after 34 h; despite the late start, maternal and fetal well-being was maintained, suggesting the utility of therapy outside the window, which may be explained by the permanence of paraquat in the blood [8]. The joint use of Hemoadrorption with CVVHD has been shown to decrease the rates of multiple organ dysfunction, acute respiratory distress syndrome, and mortality compared to emoadsoption [9], [10].

This case has the limitation of not obtaining an objective confirmation, such as the measurement of paraquat in urine or blood; however, emphasis was placed on the clinical history, and the toxicity was verified through photographs. This success story is published with the intention of sharing the benefits of new extracorporeal support therapies in an unusual case, gaining a better understanding of paraquat toxicokinetics in specific cases, which has important clinical significance and will guide better treatment strategies.

## 4 Conclusion

We present the case of a patient in the second trimester of pregnancy with severe paraquat poisoning who was treated with the HA-230 Hemoadsorption cartridge followed by continuous venovenous hemodiafiltration for 120 h, in addition to therapy with methylprednisolone and cyclophosphamide. Maternal and fetal well-being can be maintained during hospitalization and at the end of pregnancy and is shared as an alternative treatment.

## Funding

This research did not receive any specific grants from funding agencies in the public, commercial, or not-for-profit sectors.

## Statement of ethics

Informed consent was obtained from the patient and approval of the institutional ethics committee with internal code 00118HUSJ-CI, endorsement No. 29-2023.

## CRediT authorship contribution statement

**David A. Ballesteros:** Conceptualization, Methodology. **Daniel R. Santiago:** Data curation, Writing – original draft. **María E. Barrera:** Data curation, Writing – original draft. **Andrea C. Mantilla:** Writing – review & editing.

## Declaration of Competing Interest

The authors declare that they have no known competing financial interests or personal relationships that could have appeared to influence the work reported in this paper.

## Data Availability

Data will be made available on request.
